# Sustainable radiology in South Africa

**DOI:** 10.4102/sajr.v28i1.3065

**Published:** 2024-11-28

**Authors:** Maya Patel

**Affiliations:** 1 Department of Radiology, School of Medicine, Faculty of Health Sciences, University of the Witwatersrand, Johannesburg, South Africa

Radiation safety dictates that we apply the ‘as low as reasonably achievable’ principle to minimise radiation. The converse is true for sustainability, where we need to do ‘as much as reasonably impactful’ to maximise our efforts towards protecting the environment, confronting climate change and preserving healthy societies.

Sustainable medical imaging has been a trending topic in the global radiology fraternity recently, but awareness of its importance and actions to impede further environmental harm are taking effect at different rates across the world. The healthcare sector has been tagged as a significant contributor to the carbon-footprint and waste production, and within this sector, clinical radiology is one of the more responsible divisions.

Examples of energy burdens in radiology that contribute to greenhouse gases include manufacture and transport, high-energy consuming equipment, cooling systems, computer workstations, considerable data generation and data storage. Excessive waste production in radiology is instrumental in environmental pollution and stems from single-use consumables, particularly for imaging-guided procedures, and the widespread use of contrast media for both CT and MRI.^1,2,3^ Recent studies have documented contrast media not only in hospital sewage water but also in surface, ground and drinking water with potentially environmentally toxic by-products.^4^ The problems are extensively listed and contemporaneously, solutions for sustainability have been proffered.

Increasing awareness, education and training are probably the starting points and hopefully this editorial will promote further discussion and reading around the topic. Dedicated task force teams, expert opinions, policies, collaborations and leaders are indispensable, but simple actions can be implemented without directives. These include powering off imaging equipment when not in use, decreasing machine idle times by scheduling scans, tailoring scan protocols, and abiding by guidelines for imaging tests. Machines can be upgraded rather than replaced and plans can be made towards the use of renewable energy sources. Recycling is pivotal to non-regulated waste management, especially when the recycled materials can be repurposed for other uses, creating a circular economy rather than a linear route to landfill. Indications for the usage of contrast need to be supervised and multidose contrast injector systems can be used instead of single-dose packaging.^1,2,3^ Changes made can be beneficial to the healthcare sector, the environment and to the health of individuals in society.

In their systematic review published in 2023, Anudjo et al.^5^ found that studies published on the topic of sustainability in clinical radiology and radiotherapy were conducted in Europe, North America and Australia. According to data from 2022, South Africa is the highest producer of CO_2_ emissions and greenhouse gases on the African continent, but research specific to the medical sector is lacking. In a quantitative study published by Chinene et al.^6^ in 2024, in their sample of 216 radiographers from Zimbabwe and Zambia, 44% noted that education on sustainability was lacking, and approximately 50% reported no sustainable practices in their departments. The main barriers mentioned were a lack of priority for sustainability among leadership, a lack of incentives for sustainability and a lack of partnerships between suppliers and consumers for sustainability. Similarly, South Africa is faced with a multitude of pressing challenges and prioritising and incentivising sustainability over issues such as poverty, inequality, unemployment, crime and provision of public services, is not an easy task.

In South Africa, imaging services vary between public and private healthcare facilities, and older machines are more likely to be more energy-consuming than newer models. Economic constraints limit the procurement of sustainability-friendly equipment like dual energy CT where the number of scan phases can be reduced. Although digital imaging has replaced analogue imaging, digital hospital systems are lacking and several departments are not paperless, adding to the burden of waste. In addition, only a small percent of South Africans recycle, with up to 90% of waste ending up in landfills, which have a finite capacity and pose health risks.

While developing countries are overburdened and resource-limited, they are also more likely to be afflicted by the consequences of climate change and pollution on their environment, infrastructure and health. The need for change is clear and this global crisis is not specific to developed or developing countries, overpopulated countries or medical professionals. Progress can only occur once we begin; therefore, start today to learn, collaborate and perform purposeful actions that will allow you to be as impactful as possible within your own circumstances.
